# Transcriptomic Response of Purple Willow (*Salix purpurea*) to Arsenic Stress

**DOI:** 10.3389/fpls.2017.01115

**Published:** 2017-06-28

**Authors:** Aymeric Yanitch, Nicholas J. B. Brereton, Emmanuel Gonzalez, Michel Labrecque, Simon Joly, Frederic E. Pitre

**Affiliations:** ^1^Institut de Recherche en Biologie Végétale, University of MontrealMontréal, QC, Canada; ^2^Canadian Centre for Computational Genomics, C3G Montreal Node, McGill University and Genome Quebec Innovation CentreMontréal, QC, Canada; ^3^Montreal Botanical GardenMontréal, QC, Canada

**Keywords:** *Salix*, arsenic, phytoremediation, transcriptomics, abiotic stress tolerance, RNA-seq, trace elements

## Abstract

Arsenic (As) is a toxic element for plants and one of the most common anthropogenic pollutants found at contaminated sites. Despite its severe effects on plant metabolism, several species can accumulate substantial amounts of arsenic and endure the associated stress. However, the genetic mechanisms involved in arsenic tolerance remains obscure in many model plant species used for land decontamination (phytoremediation), including willows. The present study assesses the potential of *Salix purpurea* cv. ‘Fish Creek’ for arsenic phytoextraction and reveals the genetic responses behind arsenic tolerance, phytoextraction and metabolism. Four weeks of hydroponic exposure to 0, 5, 30 and 100 mg/L revealed that plants were able to tolerate up to 5 mg/L arsenic. Concentrations of 0 and 5 mg/L of arsenic treatment were then used to compare alterations in gene expression of roots, stems and leaves using RNA sequencing. Differential gene expression revealed transcripts encoding proteins putatively involved in entry of arsenic into the roots, storage in vacuoles and potential transport through the plant as well as primary and secondary (indirect) toxicity tolerance mechanisms. A major role for tannin as a compound used to relieve cellular toxicity is implicated as well as unexpected expression of the cadmium transporter CAX2, providing a potential means for internal arsenic mobility. These insights into the underpinning genetics of a successful phytoremediating species present novel opportunities for selection of dedicated arsenic tolerant crops as well as the potential to integrate such tolerances into a wider *Salix* ideotype alongside traits including biomass yield, biomass quality, low agricultural inputs and phytochemical production.

## Introduction

Arsenic is a trace element recognized as a worldwide contaminant and health hazard (Martinson and Reddy, 2009). Natural geologic activity is thought to be the main source of global arsenic pollution but highly contaminated sites are generally related to anthropogenic activities such as agriculture, mining, as well as the use of arsenic in electronics or as a wood preservative (Mandal and Suzuki, 2002).

Arsenic, a metalloid element, is highly toxic to microorganisms, plants and animals (Kaise et al., 1985). In animals, arsenic is absorbed through drinking water and food (Duxbury et al., 2003; Henry et al., 2013) and once ingested, arsenic enters in cells and can generate oxidative damage to DNA leading to a well-documented carcinogenic effect (Ng, 2005; Cohen et al., 2013). Arsenic toxicity to plants has been extensively studied (Woolson, 1973; Carbonell et al., 1998; Tripathi et al., 2007) and triggers symptoms such as root growth inhibition, desiccation or death in non-tolerant plants (Meharg and Hartley-Whitaker, 2002).

The chemical state of arsenic is dependent on soil conditions such as pH, organic content and redox potential (Zhao et al., 2009; Bolan et al., 2015). Arsenate (As^V^) appears to be the most abundant form in aerobic conditions, while arsenite (As^III^) is the major chemical state of this metalloid under a reducing environment (Mandal and Suzuki, 2002). The chemical similarity of the arsenate ion (${\text{AsO}}_{4}^{3-}$) and phosphate creates competition between both compounds and once inside the cell cytoplasm, arsenate can replace phosphate in respiration processes, disrupting cellular metabolism (generating ADP-As in place of ATP) (Meharg, 1994). Arsenite (As^III^) toxicity is predominantly due to its high reactivity with sulfhydryl groups present in a broad range of metabolic enzymes (Dhankher et al., 2002).

The decontamination of soil and water contaminated with arsenic presents a challenge for environmental rehabilitation. The traditional decontamination technique is to excavate the soil and dispose of it in a protected landfill. Phytoremediation is a potential environmentally sustainable alternative to this physical (excavation and disposal to landfill) or chemical (chelation, thermic desorption, soil washing, etc.) decontamination processes (Mench et al., 2009; Wan et al., 2016) that relies on natural plants properties that, while taking up water and nutrients from soil, can also import pollutants into their tissues (Pulford and Watson, 2003).

Substantial arsenic phytoremediation research has focused on hyperaccumulating plants that can concentrate high amount of metal per gram of tissue (Ma et al., 2001; Wang et al., 2002; Poynton et al., 2004). However, hyperacumulators are generally limited by a low biomass production. One of the most well-studied and effective plants which concentrate arsenic is the Chinese Brake fern (*Pteris vittata*) that can tolerate 1.5 mg.g^−1^ of soil arsenic and accumulate up to 150 mg.g^−1^ arsenic in its tissues (the majority being above-ground in fronds) (Ma et al., 2001; Meharg and Hartley-Whitaker, 2002). In contrast, willows (*Salix* sp.) generally take up a lower concentration of trace elements per gram of tissue but, due to their higher biomass productivity, can extract comparable net amounts of pollutant (Purdy and Smart, 2008). *Salix* is a diverse genus with about 450 species (Lauron-Moreau et al., 2015), some of which have been shown to have potential for phytoremediation processes such as *Salix viminalis* and *Salix purpurea* (Vollenweider et al., 2006; Mleczek et al., 2010; Cloutier-Hurteau et al., 2013; Desjardins et al., 2015; Grenier et al., 2015). *Salix* spp. response to metal contamination has predominantly investigated cadmium (Cd) and zinc (Zn). For instance, *Salix caprea* can accumulate considerable amounts of Zn and Cd in their aboveground organs (Robinson et al., 2000; Dos Santos Utmazian et al., 2007; Chen et al., 2013). However, a study performed by Purdy and Smart (2008) has shown that some willow species can also uptake arsenic, suggesting they could also have phytoremediation utility on arsenic contaminated soils.

Plant gene expression response to arsenic presence has previously been described in species such as rice (*Oriza sativa*) (Ma et al., 2007; Li et al., 2009; Zhao et al., 2010; Song et al., 2014), velvet grass (*Holcus lanatus*) (Bleeker et al., 2006), *Arabidopsis thaliana* (Catarecha et al., 2007; Kamiya et al., 2009) and Chinese Brake fern (*Pteris vittata*) (Ellis et al., 2006). Several transporters have been shown to interact with arsenic to transport it across plasma membrane. High affinity phosphate transporters appear to be involved in arsenate entry (Catarecha et al., 2007), while silicon transporters (such as Lsi1) transport arsenite and methylated forms of arsenic (Li et al., 2009; Zhao et al., 2010). Once in root cells, arsenate could be reduced to arsenite by a CDC-25 phosphatase (Bleeker et al., 2006; Duan et al., 2007). Then arsenite can form a complex with phytochelatin (Hartley-Whitaker et al., 2001) and transported by ABC transporters (Song et al., 2010) to be stored in the vacuole in order to prevent cell damage.

Little research into *Salix* gene expression in response to contaminants has been undertaken to date, but typical responses are likely to involve radical oxygen species (ROS) production (Dietz et al., 1999) and expression of common detoxification mechanisms, such as the glutathione pathway(Gonzalez et al., 2015). In this study, *Salix purpurea* are grown hydroponically under different arsenic concentrations to assess arsenic accumulation and alterations to gene expression in order to affirm if these common detoxification mechanisms are indeed a general strategy employed as well as to discover any less common underpinning genetics of arsenic tolerance in willow.

## Materials and methods

### Hydroponic experiment

*Salix purpurea* cv. ‘Fish Creek’ stem cuttings of 20 cm length were established under hydroponic conditions in tanks (15 × 25 × 12 cm = 3.8 L) containing 0.25x of Hoagland solution with an aeration system to prevent lack of oxygen (Durell, 1941; Moreno-Jiménez et al., 2010). Pumps providing 4 liters of air per minute were used as recommended by Durell (1941). After 2 weeks of growth, 64 cuttings were exposed to one of four levels of arsenic contamination: 0, 5 mg/L (67 μM), 30 mg/L (400 μM), and 100 mg/L (1335 μM) (element concentration, added as sodium heptahydrate arsenate Na_2_HAsO_4_.7H_2_O; 0, 21 mg/L (67 μM), 125 mg/L (400 μM), 416 mg/L (1335 μM) of salt, respectively). Although arsenate (As^V^) was the applied species, the term arsenic is used unless specified as the relative proportions of different species *in planta* was not determined. The treatment application was randomized and the volume of each tank was maintained to a constant 3.8 L over the 2 weeks by addition of water alone. Consequently, the total amount of arsenic applied was 0 mg, 19 mg, 114 mg and 380 mg respectively.

Plants were cultivated under controlled conditions: 18–25°C with a 18 h light/6 h dark photoperiod (Purdy and Smart, 2008) under light intensity of 500 μmol.m^−2^. A total of 16 tanks were used and distributed in 4 experimental blocs (4 arsenic concentrations × 4 blocks). Of the 4 plants per tank: 1 plant was sampled for RNA extraction and sequencing 2 weeks after treatment application, and 1 plant was used for non-destructive chlorophyll content and stomatal conductance assessment. Tissue collected for RNA analysis (from roots, stems and leaves) was immediately flash frozen using liquid nitrogen and stored at −80°C until extraction. Chlorophyll content was monitored using an “atLEAF+” chlorophyll meter (FT Green LLC, DE, USA). Stomatal conductance was recorded with a leaf porometer (Decagon Devices Inc., WA, USA). Both chlorophyll content and stomatal conductance were estimated following manufacturer's instructions twice a week between 10:00 and 12:00. One plant per block was destructively harvested for biomass yield and arsenic accumulation measurements at the beginning of the treatment, after 7 days and 14 days. Biomass production was evaluated destructively by weighing fresh roots, stems and leaves separately. Arsenic quantification in each plant tissue was performed using an inductively coupled plasma mass spectrometer (ICP-MS) at AGAT Laboratories [specializing in laboratory analysis and accredited by the Standards Council of Canada (SCC)]. Results from the four treatments were compared by one-way analysis of variance (ANOVA) and pairwise comparisons with Tukey's HSD test (α = 0.05).

### Transcriptomic analyses

All frozen tissues were ground to a powder using a mortar and pestle. Total RNA isolation was then performed following a hexadecyltrimethyl ammonium bromide (CTAB) protocol (Chang et al., 1993; Gambino et al., 2008) using 100 mg of tissue. RNA integrity was assessed with a Bioanalyzer RNA 6000 Nano Kit (RIN > 9; Agilent, Santa Clara, CA, USA).

Sequencing was performed on root, stem and leaf tissue for control and plants grown in 5 mg/L arsenic (due to growth inhibition at higher concentrations). Sequencing libraries were produced at Genome Quebec Innovation Centre using the TruSeq 100 bp paired-ends kit (Illumina® TruSeq® RNA Sample Preparation Kit), which included a polyA mRNA purification step and a random hexamer cDNA synthesis. Samples were sequenced using an Illumina HiSeq 2000 sequencing platform. Reads were filtered using Trimmomatic (Lindgreen, 2012). Reads under 40 bp after filtering were discarded. A *de novo* transcriptome was assembled using Trinity software with default parameters (Grabherr et al., 2011; Haas et al., 2013) and contigs shorter than 200 bp were removed. Contig abundance was estimated and normalized using Bowtie2 (Langmead and Salzberg, 2012) and eXpress (Trapnell et al., 2012) using default parameters resulting in an average mapping rate of 96% across all samples. Differential gene expression between treatment was tested with EBSeq (Leng et al., 2013) at default parameters with a false discovery rate (FDR) set to 5% with significance identified and expressed as posterior probability differential expression (PPDE) greater or equal to 0.95.

Annotation of differentially expressed contigs was performed following Gonzalez et al. (2015) using *Salix purpurea* 94006 reference Genome (*Salix purpurea* v1.0, DOE-JGI) as well as three protein databases: nr, Swissprot, Trembl. Best hits were selected based on the highest bitscore. Gene ontology (PANTHER - Protein ANalysis THrough Evolutionary Relationships) was used to speculate at an overview of general transcriptome function (Thomas et al., 2003; Mi et al., 2007). Using the Panther analysis tool (http://www.pantherdb.org/), an overrepresentation test was performed to identify panther terms that were more or less represented (α = 0.05 for statistical tests) in the transcriptome of arsenic treated plants (Mi et al., 2013).

## Results

### Arsenic uptake and physiological response to treatment

**Figure 1:**
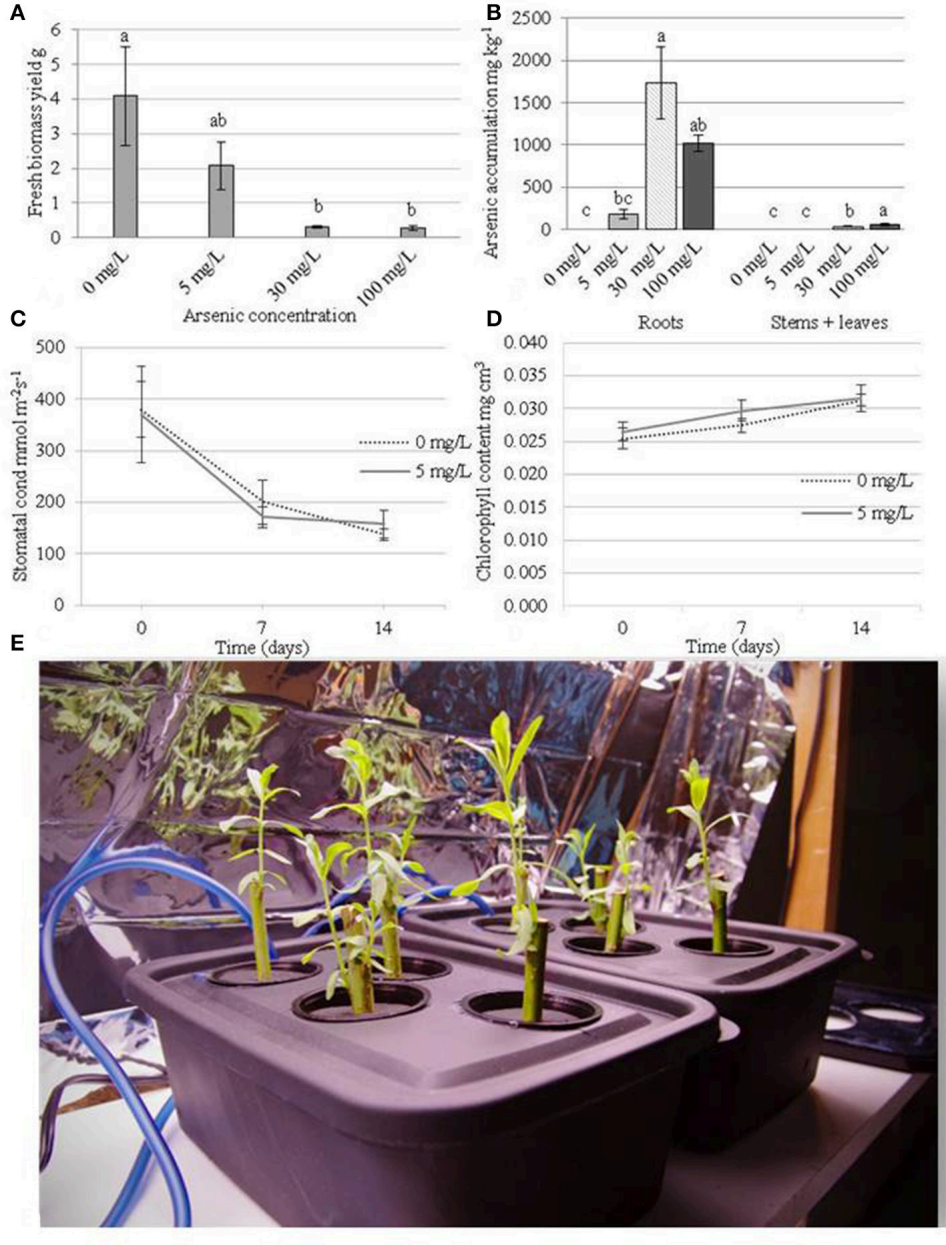
Physiological measurements of the plants during arsenic exposure. Plants were cultivated for 14 days before treatment with arsenic concentrations of 0, 5, 30, or 100 mg/L. **(A)** Fresh weight biomass yield (g) after 14 days of treatment (28 days of growth). **(B)** Arsenic accumulation in organs after 14 days of treatment. **(C)** Chlorophyll content mg.cm^3^ (day 0 represents treatment application date). **(D)** stomatal conductance mmol.m^−2^s^−1^ (day 0 represents treatment application date). Error bars represent standard error (*n* = 4 blocks). Tukey's Honestly Significant Difference (α = 0.05) is represented by lettering. **(E)** a photograph of the hydroponic tanks (15 × 25 × 12 cm = 3.8 L).

The 30 and 100 mg/L arsenic treatments reduced biomass yields by 92.7 and 93.4% respectively when compared to control plants (Figure 1A) after 2 weeks of treatment. The lowest arsenate treatment of 5 mg/L also had substantial reduction in biomass of 49.1% compared to control plants (albeit not significant using Tukey's HSD *p* > 0.05). At a concentration of 5 mg/L of arsenic, treated plants accumulated up to 183 mg/Kg arsenic in their roots while the level was below detection limit (e.g., <5 mg/Kg) in the aboveground organs (Figure 1B). Plants exposed to 30 mg/L arsenic accumulated a concentration of 1,731 mg/Kg in their roots (195 mg total arsenic) and 32 mg/Kg (6 mg total arsenic) in their aboveground tissues. At the most concentrated condition (100 mg/L), plants showed severe necrotic symptoms after 7 days of treatment but were able to accumulate 1,015 mg/Kg arsenic in their roots (134 mg total arsenic) and 841 mg/Kg arsenic in their stems and leaves (836 mg total arsenic).

Plant transpiration rate, as measured by stomatal conductance, and chlorophyll content could not be measured at 30 and 100 mg/L arsenic treatments due to the extent of plant necrosis. Transpiration rate did not differ significantly between control plants (average 239 mmol.m^−2^.s^−1^) and the 5 mg/L treatment (average 231 mmol.m^−2^.s^−1^) (Figure 1C). Similarly, chlorophyll content did not differ significantly between control plants and the 5 mg/L arsenic treatment (Figure 1D).

### Arsenic treatment transcriptome

A total of 451,706 contigs were assembled from 24 RNA samples extracted from roots, stems and leaves of 4 treated and 4 control plants. Transcript length averaged 1,647 bp (N50: 2966 bp) with a mean GC content of 40%. Across all tissues, 10,613 contigs were identified as differentially expressed (2.35%). Of these, 86.7% were best annotated as *Salix* in origin, while 5.7% were best annotated from non-salix organisms (henceforth assumed to be transcripts) and 7.6% had no confident BLASTx hit in either NCBI nr, SwissProt, TrEMBL or the *Salix purpurea* 94006 genome (no hit, bitscore < 50 or *e* > 10^−4^, classified here as unknown).

#### Differential expression in roots

**Figure 2:**
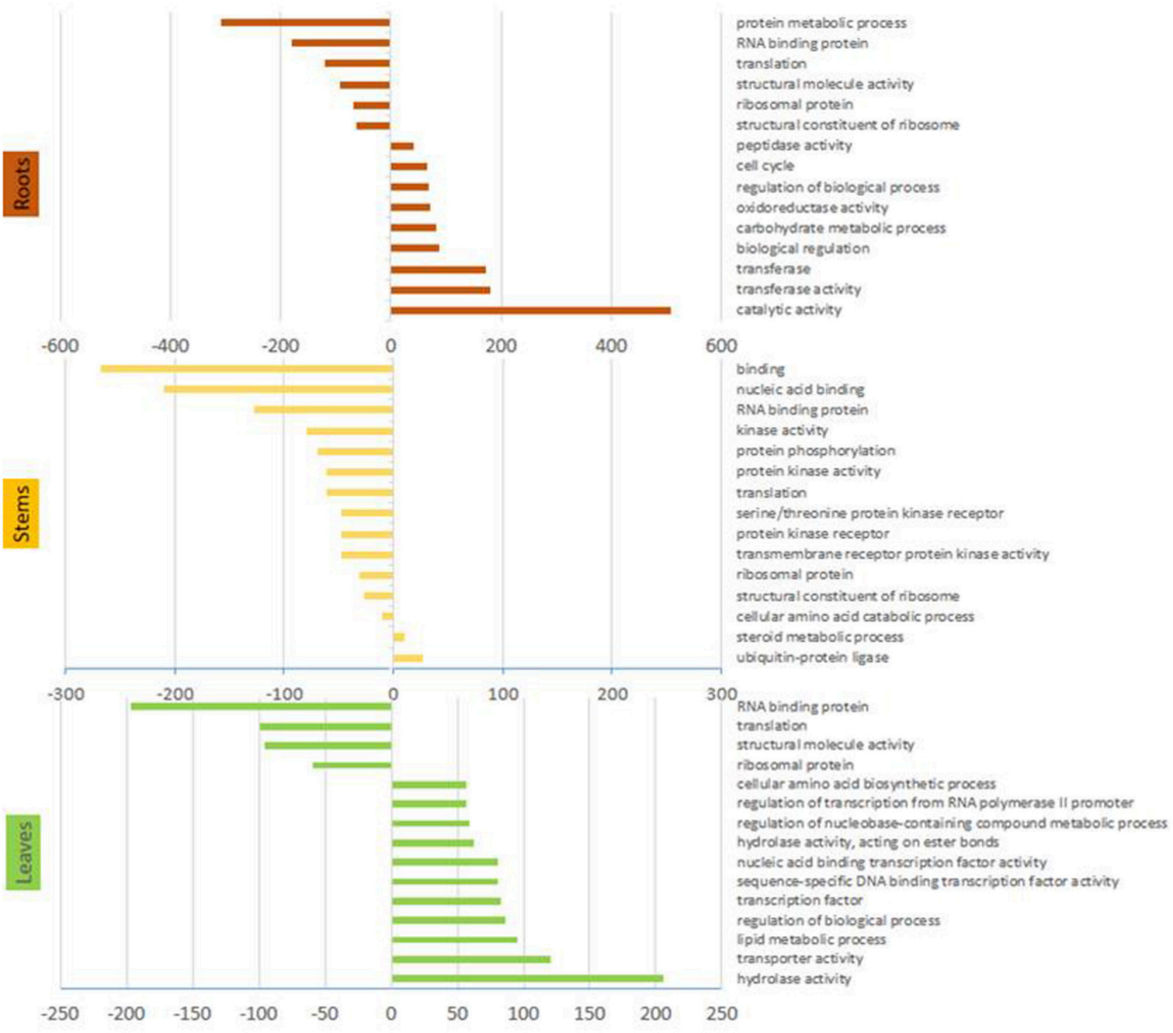
Gene ontology terms. Results from the statistical overrepresentation analysis showing significantly more and less represented Panther terms from organs of arsenic treated plants. Only the 15 most abundant terms are shown. Panther terms for each DE gene are given in supplementary file 1 if present.

Gene ontology analyses revealed that the most represented ontology category among DE genes in arsenic treated plants was catalytic activity, followed by transferase activity and biological regulation, while the most down-regulated categories included protein metabolic processes and RNA binding (Figure 2).

Variation in transcripts encoding transporter proteins such as phosphate transporters and aquaporins could be predicted upon entry of arsenic into the roots. Transcripts encoding the phosphate transporter PHO1 (SapurV1A.0063s0550.x.p) were indeed up-regulated (1.70 fold) in roots of arsenic treated trees (Table 1). Three transcripts encoding the aquaporin NIP1.1 (SapurV1A.0029s0170.x.p) were also up-regulated in the presence of arsenic (3.13, 4.76, and 2.49 fold higher respectively) with one (comp88567_c0_seq20) being in very high normalized abundance, 34.85 Fragments Per Kilobase per Million reads mapped (FPKM). Conversely, the aquaporin TIP2 (SapurV1A.0805s0180) was down-regulated (7.34 fold).

**Table 1:** A selection of differentially expressed genes in roots (all genes have a posterior probability of being differentially expressed >0.95). The full list is available in Supplementary File 1.

***De novo* assembly**	**Mean FPKM**			**Fold**	**Salix purp V1.0**	**Annotation**
**Contig id**	**Control**	**Arsenic**	**Regulation**	**Change**	**Protein ID**	**Description**
**TRANSPORTERS**						
*Phosphate transporter*						
comp94662_c0_seq9	1.51	2.57	+	1.70	SapurV1A.0063s0550.2.p	Phosphate transporter PHO1-like protein
*NIP1.1*						
comp88567_c0_seq20	0.15	0.70	+	4.77	SapurV1A.0029s0170.1.p	Aquaporin NIP1.1
comp88567_c0_seq18	0.41	1.29	+	3.14	SapurV1A.0029s0170.1.p	Aquaporin NIP1.1
comp88567_c0_seq24	14.03	34.85	+	2.48	SapurV1A.0029s0170.1.p	Aquaporin NIP1.1
*TIP2*						
comp83540_c0_seq2	2.04	0.28	−	7.34	SapurV1A.0805s0180.1.p	Aquaporin TIP2
*CAX2*						
comp93893_c1_seq57	0.05	0.24	+	5.05	SapurV1A.1071s0020.11.p	Vacuolar cation/proton exchanger 2
comp93893_c1_seq50	1.90	8.74	+	4.61	SapurV1A.0338s0120.1.p	Vacuolar cation/proton exchanger 2
*ABC transporters*						
comp90450_c0_seq1	0.02	0.37	+	22.39	SapurV1A.0084s0020.1.p	White-brown-complex ABC transporter
comp88754_c0_seq6	0.15	0.84	+	5.58	SapurV1A.0771s0060.1.p	Multidrug exporter, ABC transporter
comp77006_c0_seq14	0.27	0.75	+	2.75	SapurV1A.1733s0060.2.p	ABC1-domain protein
comp79446_c0_seq6	0.51	1.27	+	2.49	SapurV1A.0001s1760.1.p	ABC(ATP-binding) family transporter
comp93840_c0_seq5	1.61	3.89	+	2.42	SapurV1A.0202s0010.1.p	Multidrug resistance protein ABC transporter
comp91076_c0_seq38	0.51	1.12	+	2.21	SapurV1A.0317s0290.1.p	ABC transporter B
comp91858_c0_seq22	4.24	6.81	+	1.61	SapurV1A.1189s0020.1.p	Multidrug resistance pump protein
comp88754_c0_seq19	1.57	2.34	+	1.49	SapurV1A.0438s0120.1.p	Multidrug exporter, ABC transporter
comp94792_c2_seq58	0.14	0.00	−	54.58	SapurV1A.0326s0140.1.p	ABC transporter G family protein
comp36900_c0_seq1	0.44	0.01	−	43.43	SapurV1A.1188s0080.1.p	ABC transporter
comp84963_c0_seq2	10.39	0.57	−	18.32	SapurV1A.1334s0010.1.p	ABC transporter, phospholipid flippase
comp90794_c0_seq50	0.56	0.03	−	17.32	SapurV1A.1019s0010.1.p	ABC transporter
comp93051_c1_seq45	0.45	0.03	−	14.18	SapurV1A.0028s0110.1.p	Multidrug resistance protein ABC transporter
comp94792_c2_seq63	1.08	0.08	−	14.06	SapurV1A.0326s0140.1.p	ABC transporter G family protein
comp94792_c2_seq32	1.00	0.08	−	13.34	SapurV1A.0326s0140.1.p	ABC transporter G family protein
comp94792_c2_seq37	0.18	0.01	−	13.34	SapurV1A.0326s0140.1.p	ABC transporter G family protein
comp92573_c0_seq2	0.17	0.02	−	9.60	SapurV1A.1230s0070.1.p	ABC transporter family B, PpABCB26
comp92573_c0_seq10	0.25	0.04	−	6.64	SapurV1A.1230s0070.1.p	ABC transporter B protein PpABCB26
comp89364_c0_seq6	0.84	0.14	−	5.90	SapurV1A.0902s0030.1.p	ABC1
comp95190_c0_seq25	0.62	0.22	−	2.83	SapurV1A.0479s0010.1.p	Multidrug resistance protein ABC transporter
comp94365_c1_seq15	1.54	0.83	−	1.85	SapurV1A.0169s0040.1.p	ABC transporter
comp89676_c0_seq3	0.74	0.43	−	1.71	SapurV1A.0252s0220.1.p	Multidrug resistance protein ABC transporter
comp87484_c0_seq75	0.78	0.51	−	1.53	SapurV1A.0153s0550.1.p	Multidrug resistance protein ABC transporter
comp94768_c0_seq5	4.77	3.14	−	1.52	SapurV1A.0038s0650.1.p	Multidrug exporter, ABC transporter
comp95190_c0_seq28	1.56	1.08	−	1.45	SapurV1A.0479s0010.1.p	Multidrug resistance protein ABC transporter
comp94768_c0_seq2	2.55	1.84	−	1.39	SapurV1A.0038s0650.1.p	Multidrug exporter, ABC transporter
comp95238_c0_seq3	3.34	2.72	−	1.23	SapurV1A.0294s0380.1.p	Multidrug exporter, ABC transporter
**ARSENIC METABOLISM**						
*Tyrosine phosphatase*						
comp93933_c0_seq5	1.26	2.61	+	2.08	SapurV1A.0142s0310.2.p	Tyrosine phosphatase
comp95075_c2_seq9	1.67	0.85	−	1.97	SapurV1A.0243s0430.1.p	Tyrosine phosphatase
*GS*						
comp94552_c0_seq13	4.18	9.31	+	2.23	SapurV1A.1124s0080.1.p	Glutathione synthetase
comp94552_c0_seq17	46.34	93.13	+	2.01	SapurV1A.1124s0080.1.p	Glutathione synthetase
comp94552_c0_seq22	3.82	5.87	+	1.54	SapurV1A.1124s0080.1.p	Glutathione synthetase
*PCS*						
comp91524_c1_seq2	0.09	0.96	+	10.33	SapurV1A.1703s0010.4.p	Phytochelatin synthetase-like protein
comp91524_c1_seq9	0.38	2.26	+	6.01	SapurV1A.1703s0010.4.p	Phytochelatin synthetase-like protein
comp92922_c0_seq2	0.42	1.36	+	3.22	SapurV1A.0160s0210.1.p	Phytochelatin synthase
*SAM*						
comp82683_c0_seq2	0.11	0.53	+	4.98	SapurV1A.0015s0590.1.p	S-adenosyl-L-methionine:carboxyl MT
comp91039_c3_seq21	0.16	0.52	+	3.34	SapurV1A.0335s0120.2.p	S-adenosylmethionine-dependent MT
comp91039_c3_seq16	1.57	4.72	+	3.01	SapurV1A.0335s0120.2.p	S-adenosylmethionine-dependent MT
comp91039_c3_seq41	0.34	1.01	+	2.94	SapurV1A.0335s0120.2.p	S-adenosylmethionine-dependent MT
comp85603_c0_seq18	0.90	2.24	+	2.49	SapurV1A.0447s0070.1.p	S-adenosyl-L-methionine-dependent MT
comp89150_c0_seq9	1.78	3.40	+	1.91	SapurV1A.0011s0500.1.p	S-adenosylmethionine-dependent MT
comp82009_c0_seq4	6.42	10.63	+	1.66	SapurV1A.0515s0050.1.p	S-adenosyl-L-methionine-dependent MT
comp91683_c1_seq5	0.15	0.00	−	41.04	SapurV1A.0176s0200.1.p	S-adenosyl-L-methionine-dependent MT
comp95439_c0_seq35	0.36	0.06	−	6.28	SapurV1A.0386s0140.1.p	S-adenosyl-L-methionine:SA carboxyl MT
comp95439_c0_seq22	0.09	0.02	−	5.03	SapurV1A.0386s0140.1.p	S-adenosyl-L-methionine:SA carboxyl MT
comp94259_c1_seq52	0.55	0.21	−	2.55	SapurV1A.0214s0280.2.p	S-adenosyl-L-methionine-dependent MT
comp95439_c0_seq24	3.98	2.37	−	1.68	SapurV1A.0386s0140.1.p	S-adenosyl-L-methionine:SA carboxyl MT
comp95439_c0_seq8	3.82	2.51	−	1.52	SapurV1A.0386s0140.1.p	S-adenosyl-L-methionine:SA carboxyl MT
*ACC synthase*						
comp82878_c0_seq1	0.48	0.95	+	1.97	SapurV1A.2160s0020.1.p	1-aminocyclopropane-1-carboxylate synthase
*EIN*						
comp94650_c1_seq1	0.14	1.36	+	9.74	SapurV1A.0070s0670.1.p	Ethylene insensitive 3 class TF

Genes known to be involved in arsenic reduction could also be expected to be differentially expressed in the roots to decrease cellular toxicity. Two transcripts of CDC25-like tyrosine phosphatase, an arsenate reductase, were differentially expressed in roots; one up-regulated after arsenic treatment (SapurV1A.0142s0310) (2.08 fold) and the other down-regulated (SapurV1A.0243s0430) (1.97 fold) (Table 1). The first step of phytochelatin (PC) production involves gamma-glutamylcystein synthetase (ɤECS) to generate gamma-glutamylcystein from cysteine. ɤECS in roots were not differentially expressed between arsenic and control plants; however, expression levels of three glutathione synthase (GS) transcripts encoding the same protein (SapurV1A.1124s0080) were found to be in the most abundant transcripts in roots exposed to arsenic (one at 93.13 FPKM; Table 1 and Supplementary File 1). Phytochelatin production from glutathione (GSH) involves phytochelatin synthetase (PCS). PCS transcription was also up-regulated in plants exposed to arsenic; three transcripts encoding two PCS proteins (SapurV1A.1703s0010.x.p and SapurV1A.0160s0210.x.p) were up-regulated in roots of treated trees.

Once complexed to phytochelatin, arsenite could be taken up by ABC transporters and stored in the vacuole. A large number of transcripts encoding ABC transporters were differentially expressed; eight transcripts were up-regulated in arsenic treated trees while 19 transcripts were down-regulated (Table 1). Two vacuolar cation/proton exchanger 2 (CAX2) transcripts, encoding two different proteins (SapurV1A.0338s0120.x.p and SapurV1A.1071s0020.x.p), were both up-regulated in arsenic treated roots (respectively 4.61 and 5.05 fold higher) (Table 2A).

**Table 2:** A selection of differentially expressed genes in stems (all genes have a posterior probability of being differentially expressed > 0.95). The full list is available in Supplementary File 1.

***De novo* assembly**	**Mean FPKM**			**Fold**	**Salix purp V1.0**	**Annotation**
**Contig id**	**Control**	**Arsenic**	**Regulation**	**Change**	**Protein ID**	**Description**
**TRANSPORTERS**						
*Silicon transporter*						
comp87154_c0_seq2	1.31	2.11	+	1.61	SapurV1A,1225s0080,1,p	Silicon transporter
*Cation vacuolar*						
comp66704_c0_seq4	0.17	0.58	+	3.39	SapurV1A,0338s0120,1,p	Vacuolar cation/proton exchanger 2
comp92964_c1_seq3	10.06	3.47	−	2.90	SapurV1A,0619s0210,1,p	Vacuolar cation/proton exchanger
comp47917_c0_seq1	9.51	3.45	−	2.76	SapurV1A,0619s0210,1,p	Vacuolar cation/proton exchanger
**RIBOSOMAL PROTEIN**						
comp93888_c0_seq29	0.01	0.17	+	13.89	SapurV1A,0013s1150,1,p	50S ribosomal protein L5
comp89712_c0_seq27	0.51	1.02	+	2.00	SapurV1A,2715s0010,1,p	Ribosomal protein L15
comp90500_c1_seq47	0.38	0.03	−	13.44	SapurV1A,1939s0020,1,p	60S ribosomal protein L23a
comp89290_c0_seq18	0.32	0.02	−	13.14	SapurV1A,0032s0190,1,p	50S ribosomal protein L21
comp94061_c0_seq84	0.09	0.01	−	11.55	SapurV1A,1667s0040,1,p	Ribosomal protein L18
comp92144_c0_seq9	1.26	0.26	−	4.79	SapurV1A,0470s0210,1,p	60S ribosomal protein L2
comp77477_c0_seq1	1.32	0.41	−	3.20	SapurV1A,0023s0480,1,p	Ribosomal protein S23
comp93434_c0_seq51	6.16	1.95	−	3.16	SapurV1A,0435s0080,1,p	40S ribosomal protein S3
comp49167_c0_seq4	0.39	0.14	−	2.80	SapurV1A,0205s0160,1,p	50S ribosomal protein L13
comp88461_c0_seq2	1.21	0.46	−	2.62	SapurV1A,1377s0110,1,p	50S ribosomal protein L17
comp90935_c0_seq43	45.54	17.73	−	2.57	SapurV1A,0045s0130,1,p	40S ribosomal protein S15a
comp93434_c0_seq18	14.76	6.83	−	2.16	SapurV1A,0435s0080,1,p	40S ribosomal protein S3
comp94423_c6_seq14	6.75	3.28	−	2.06	SapurV1A,0508s0040,1,p	60S ribosomal protein L6
comp90563_c0_seq4	38.69	19.56	−	1.98	SapurV1A,0101s0170,1,p	40S ribosomal protein S7
comp94786_c1_seq23	9.32	4.82	−	1.93	SapurV1A,0061s0080,1,p	60S acidic ribosomal protein P0
comp93980_c0_seq16	6.68	3.46	−	1.93	SapurV1A,0021s0480,1,p	60S ribosomal protein L23
comp92282_c0_seq18	1.35	0.71	−	1.91	SapurV1A,0231s0170,1,p	40S ribosomal protein S6
comp94958_c2_seq16	1.72	0.93	−	1.85	SapurV1A,0037s0170,1,p	40S ribosomal protein S2
comp90935_c0_seq47	100.02	56.77	−	1.76	SapurV1A,0045s0130,1,p	40S ribosomal protein S15a
comp91986_c1_seq7	3.95	2.28	−	1.73	SapurV1A,0580s0150,1,p	40S ribosomal protein S15
comp94958_c2_seq13	2.09	1.22	−	1.72	SapurV1A,0037s0170,1,p	40S ribosomal protein S2
comp88868_c2_seq4	1.28	0.75	−	1.70	SapurV1A,0517s0140,1,p	60S ribosomal protein L34
comp86140_c0_seq8	16.27	9.58	−	1.70	SapurV1A,0661s0130,1,p	40S ribosomal protein S24
comp91836_c0_seq2	9.77	6.47	−	1.51	SapurV1A,0171s0040,1,p	50S ribosomal protein L18
comp91413_c1_seq3	1.98	1.34	−	1.48	SapurV1A,0626s0140,1,p	Ribosomal protein S6
comp92282_c0_seq19	155.47	106.79	−	1.46	SapurV1A,0231s0170,1,p	40S ribosomal protein S6
comp86140_c0_seq5	267.28	190.78	−	1.40	SapurV1A,0676s0040,1,p	40S ribosomal protein S24
comp84942_c0_seq4	283.88	207.22	−	1.37	SapurV1A,0096s0140,1,p	60S ribosomal protein L17
comp91376_c0_seq3	178.10	130.91	−	1.36	SapurV1A,0621s0100,1,p	40S ribosomal protein S5
comp94019_c0_seq1	297.57	219.36	−	1.36	SapurV1A,0416s0090,1,p	40S ribosomal protein S9
comp58229_c0_seq1	201.73	152.08	−	1.33	SapurV1A,0036s0380,1,p	40S ribosomal protein S17
comp83386_c0_seq7	3.73	2.81	−	1.33	SapurV1A,0278s0080,1,p	Ribosomal protein S21
comp93206_c0_seq1	45.61	34.82	−	1.31	SapurV1A,0136s0430,1,p	50S ribosomal protein L27
**CELL WALL**						
*Cellulose biosynthesis*						
comp92704_c0_seq29	0.05	0.72	+	13.81	SapurV1A,0828s0050,1,p	Cellulose synthase A catalytic subunit 9
comp90822_c0_seq8	4.83	2.99	−	1.62	SapurV1A,2489s0010,1,p	Cellulose synthase A, catalytic subunit
comp89528_c1_seq5	2.20	0.71	−	3.11	SapurV1A,0336s0010,1,p	Cellulose synthase catalytic subunit
comp82751_c0_seq4	1.52	1.12	−	1.36	SapurV1A,0437s0060,1,p	Cellulose synthase-like protein D
FLA						
comp91090_c1_seq10	0.29	0.06	−	4.84	SapurV1A,0258s0160,2,p	Fasciclin-like arabinogalactan protein
*SPS*						
comp93322_c0_seq9	0.04	0.46	+	11.28	SapurV1A,0034s0100,1,p	Sucrose phosphate synthase
comp93322_c0_seq21	0.04	0.28	+	7.94	SapurV1A,0034s0100,1,p	Sucrose phosphate synthase
comp93322_c0_seq27	0.06	0.46	+	7.30	SapurV1A,0034s0100,1,p	Sucrose phosphate synthase
*Callose synthesis*						
comp95276_c0_seq24	0.89	0.42	−	2.12	SapurV1A,0009s0010,1,p	Callose synthase
comp95276_c0_seq70	26.36	16.84	−	1.57	SapurV1A,0009s0010,1,p	Callose synthase
*1,3-β-glucan synthesis*						
comp82416_c0_seq2	2.50	1.39	−	1.80	SapurV1A,0752s0050,1,p	1,3-beta-glucan synthase
**CROSS COMMUNICATION**						
*SAM*						
comp93789_c1_seq28	1.12	0.52	−	2.14	SapurV1A,0589s0070,1,p	S-adenosylmethionine synthase
*ACO*						
comp69294_c0_seq2	3.81	0.53	−	7.16	SapurV1A,0904s0050,1,p	1-aminocyclopropane-1-carboxylate oxidase
*Ethylene*						
comp88649_c1_seq70	0.55	0.19	−	2.87	SapurV1A,0052s0240,1,p	Ethylene receptor

S-adenosyl methionine (SAM)-dependent methyltransferase mediated arsenic methylation is thought to be a principal plant physiological detoxification process. Seven transcripts from five SAM-dependent methyltransferase genes were up-regulated in arsenate treated plants whereas six transcripts from three genes were down-regulated (Table 1). Ethylene is a phytohormone known to be involved in organs cross communication during a stress. One transcript encoding aminocyclopropane-1-carboxylate synthase (ACC synthase; SapurV1A.2160s0020.x.p), producing the ethylene precursor ACC, was up-regulated (7.95 fold higher) in response to the treatment. Transcripts associated to ethylene Insensitive factor (EIN; SapurV1A.0070s0670.x.) were also up-regulated in treated trees.

#### Differential expression in stems

Gene ontology analysis showed that 17 of 20 most abundant Panther terms identified by pooling differentially expressed genes were more abundant in control plants. The majority of these were categories associated to DNA, RNA or protein synthesis or regulation (Figure 2). The up-regulated categories comprised ubiquitin ligase (+activity) and steroid metabolism.

Transcripts encoding a silicon transporter (SapurV1A.1225s0080.x.p), previously identified as being involved in loading of methylated forms of arsenic into the stem (Li et al., 2009), were up-regulated in stem tissue from treated plants (1.61 fold higher) (Table 2). As in roots, CAX2 transcripts (SapurV1A.0338s0120.x.p), thought to be involved in arsenite sequestration into vacuole, were also found to be up-regulated in stems from treated plants (3.39 fold higher). Conversely, two others CAX transcripts encoding one *Salix* gene (SapurV1A.0619s0210) were down-regulated in stems.

Expression of transcripts encoding ribosomal proteins appeared to be consistently down-regulated in arsenic treated stems. Thirty one ribosomal transcripts were all found to be down-regulated in treated stems (Table 2). Within the cellulose biosynthesis pathway, three transcripts encoding *Salix* cellulose synthase A (CesA) were down-regulated in treated plants (SapurV1A.0437s0060.x/SapurV1A.0336s0010.x/SapurV1A.2489s0010.x), while one CesA 9 encoding transcript was up-regulated following arsenic treatment (13.81 fold higher) (SapurV1A.0828s0050.x). Abundance of a transcript encoding fasciclin-like arabinogalactan protein (FLA, SapurV1A.0258s0160.x.p), a cell wall glycoprotein, was down-regulated in stems (4.84 fold lower).

Further alterations to carbon partitioning were indicated by up-regulation of three transcripts encoding sucrose phosphate synthase (SPS) (SapurV1A.0034s0100.x.p) in treated stems (Table 2). Callose production is often thought to be involved in metal stress response and metal diffusion limitation. Two transcripts encoding callose synthase (SapurV1A.0009s0010.x.p) were down-regulated (2.12 and 1.57 fold lower) in treated stems as well as a transcript encoding β-1, 3-glucan synthase (SapurV1A.0752s0050), a mandatory enzyme for callose synthesis (1.80 fold lower).

A general pattern of down-regulation of ethylene related proteins was found in stems of plants exposed to arsenic. Transcripts encoding S-adenosylmethionine synthase (SAM synthase; SapurV1A.0589s0070.x.p), involved in the early steps of ethylene synthesis, were down-regulated (2.14 fold lower) (Table 2) as well as transcripts encoding the enzyme involved in the last step of ethylene production: aminocyclopropane carboxylate oxidase (ACO; SapurV1A.0904s0050.x.p) (7.16 fold lower) in addition to the ethylene receptor (SapurV1A.0052s0240.x.p) (2.87 fold lower). Moreover, 3 transcripts encoding an ethylene-response factor and 13 transcripts encoding an ethylene-response factor 2 were also all down-regulated in stems of treated plants (Supplementary File 1).

Salicylic acid is an important transduction signal involved in several defense mechanisms against both biotic and abiotic stresses (including metal stress). A transcript encoding salicylic acid carboxyl methyltransferase (SapurV1A.1772s0020.x.p), the enzyme involved in the last step of salicylic acid production, was up-regulated in treated stems (2.27 fold higher) (Table 2).

#### Differential expression in leaves

Gene ontology enrichment analysis indicated hydrolase and transporter activity were the most represented categories in arsenic treated leaves (Figure 2). The transporter activity was reflected in transcripts putatively involved in arsenic import from other organs that were differentially expressed in the leaves. Two transcripts encoding the phosphate transporter PHO1 (SapurV1A.0060s0410.x.p and SapurV1A.0139s0260.x.p) were up-regulated (1.85 and 1.38 fold higher respectively) in leaves from arsenic treated trees, while another PHO1 encoding transcript (SapurV1A.0063s0550.x.p) was down-regulated (4.18 fold lower) (Table 3). Similar to both root and stem differential expression, several transcripts encoding aquaporin transporters, NIP (SapurV1A.3123s0010.x.p), NIP1.1 (SapurV1A.0285s0290.x.p), TIP1 (SapurV1A.0146s0060.x.p), and SIP1 (SapurV1A.0014s1220.x.p), were up-regulated in arsenic treated plants (3.38, 1.27, and 1.45 fold higher, respectively), although a single NIP transcript was also down-regulated. A transcript encoding a boron transporter (SapurV1A.0014s0200.x.p) was also significantly up-regulated in leaves from arsenic treated plants (2.20 fold higher and in high abundance: 21.13 FPKM).

**Table 3:** A selection of differentially expressed genes in leaaves (all genes have a posterior probability of being differentially expressed > 0.95). The full list is available in Supplementary File 1.

***De novo* assembly**	**Mean FPKM**			**Fold**	**Salix purp V1.0**	**Annotation**
**Contig id**	**Control**	**Arsenic**	**Regulation**	**Change**	**Protein ID**	**Description**
**TRANSPORTERS**						
*Phosphate transporters*						
comp94906_c0_seq32	1.33	2.46	+	1.85	SapurV1A,0060s0410,1,p	Phosphate transporter 1
comp81621_c0_seq7	37.84	52.18	+	1.38	SapurV1A,0139s0260,1,p	Sodium-dependent phosphate transporter
comp94662_c0_seq31	0.80	0.19	−	4.18	SapurV1A,0063s0550,2,p	Phosphate transporter PHO1-like protein
*Boron transporter*						
comp94619_c1_seq9	9.62	21.13	+	2.20	SapurV1A,0014s0200,2,p	Boron transporter
*Aquaporins*						
comp92063_c0_seq9	5.64	19.07	+	3.38	SapurV1A,3123s0010,1,p	Aquaporin NIP domain protein
comp76373_c0_seq4	10.56	21.73	+	2.06	SapurV1A,1058s0060,1,p	Aquaporin, SIP subfamily protein
comp94505_c0_seq2	2.97	4.33	+	1.46	SapurV1A,0014s1220,1,p	Aquaporin SIP1
comp42155_c0_seq2	5.68	7.23	+	1.27	SapurV1A,0146s0060,1,p	RINT-1/TIP-1 family protein
comp91892_c0_seq6	2.27	1.11	−	2.04	SapurV1A,0835s0150,1,p	Aquaporin, NIP subfamily protein
*CAX2*						
comp93893_c1_seq42	0.83	1.76	+	2.10	SapurV1A,1071s0020,11,p	Vacuolar cation/proton exchanger 2
comp93893_c1_seq13	2.02	3.33	+	1.65	SapurV1A,1071s0020,1,p	Vacuolar cation/proton exchanger 2
comp93893_c1_seq60	36.45	48.00	+	1.32	SapurV1A,0338s0120,1,p	Vacuolar cation/proton exchanger 2
*CAX1*						
comp89654_c0_seq26	9.65	3.65	−	2.65	SapurV1A,0001s0630,1,p	Vacuolar cation/proton exchanger
comp92964_c1_seq6	10.21	4.26	−	2.40	SapurV1A,0077s0070,1,p	Vacuolar cation/proton exchanger
*ABC transporters*						
comp91866_c4_seq18	0.77	23.45	+	30.49	SapurV1A,0258s0220,1,p	ABC transporter G family protein
comp91866_c4_seq106	0.04	1.18	+	27.73	SapurV1A,0258s0220,1,p	ABC transporter G family protein
comp91866_c4_seq86	0.02	0.49	+	26.40	SapurV1A,0258s0220,6,p	ABC transporter G family protein
comp91866_c4_seq29	0.03	0.41	+	15.99	SapurV1A,0258s0220,1,p	ABC transporter G family protein
comp87641_c0_seq2	0.04	0.69	+	15.84	SapurV1A,0053s0430,1,p	ABC-type transport system protein
comp91866_c4_seq20	0.17	2.46	+	14.83	SapurV1A,0258s0220,1,p	ABC transporter G family protein
comp95281_c0_seq19	0.10	0.86	+	8.47	SapurV1A,0398s0290,1,p	ABC transporter family protein
comp90794_c0_seq42	0.15	0.77	+	5.29	SapurV1A,0546s0020,1,p	ABC transporter family protein
comp93444_c1_seq11	0.22	1.17	+	5.27	SapurV1A,0526s0020,1,p	ABC transporter family protein
comp77006_c0_seq8	0.07	0.26	+	3.70	SapurV1A,1733s0060,1,p	ABC1-domain protein
comp91866_c4_seq52	0.10	0.33	+	3.36	SapurV1A,0298s0170,1,p	ABC transporter G family protein
comp77006_c0_seq25	0.08	0.26	+	3.29	SapurV1A,1733s0060,2,p	ABC1-domain protein
comp91866_c4_seq71	1.65	5.06	+	3.07	SapurV1A,0258s0220,6,p	ABC transporter G family protein
comp95496_c1_seq34	0.52	1.36	+	2.59	SapurV1A,0025s0120,1,p	ABC transporter family
comp84707_c0_seq1	0.15	0.35	+	2.37	SapurV1A,0068s0530,5,p	ABC transporter B family protein
comp84707_c0_seq4	0.69	1.53	+	2.21	SapurV1A,0068s0530,1,p	ABC transporter family protein
comp92192_c0_seq8	0.33	0.68	+	2.04	SapurV1A,0791s0090,4,p	ABC-type transport system protein
comp92950_c1_seq42	1.69	2.86	+	1.69	SapurV1A,1105s0100,1,p	ABC transporter family protein
comp71729_c1_seq5	36.43	52.73	+	1.45	SapurV1A,0054s0480,3,p	ABC1 family protein
comp77006_c0_seq20	5.71	7.74	+	1.36	SapurV1A,1733s0060,1,p	ABC1-domain protein
comp92950_c1_seq47	0.10	0.00	−	19.61	SapurV1A,1105s0100,1,p	ABC transporter family protein
comp93743_c0_seq2	1.14	0.67	−	1.70	SapurV1A,1255s0030,1,p	ABC-type transport system protein
comp88957_c0_seq8	1.26	0.78	−	1.61	SapurV1A,0035s0100,3,p	ABC transporter F family protein
comp87641_c0_seq9	32.79	25.02	−	1.31	SapurV1A,0053s0430,1,p	ABC-type transport system protein
*PCS*						
comp94813_c0_seq53	11.71	1.36	−	8.63	SapurV1A,0546s0010,1,p	Phytochelatin synthetase-like protein
comp91524_c1_seq18	0.56	0.25	−	2.25	SapurV1A,0323s0100,1,p	Phytochelatin synthetase-like protein
**PHENYLPROPANOID PATHWAY**						
*Chorismate mutase*						
comp84798_c0_seq4	0.07	0.33	+	4.56	SapurV1A,0372s0140,1,p	Chorismate mutase
*C4H*						
comp93666_c1_seq12	0.39	4.16	+	10.76	SapurV1A,0215s0280,1,p	P450 family 73 cinnamate 4-hydroxylase
*4CL*						
comp87029_c0_seq27	0.00	0.36	+	91.87	SapurV1A,1384s0010,1,p	4-coumarate:CoA ligase
comp87029_c0_seq11	0.03	1.98	+	75.82	SapurV1A,1384s0010,1,p	4-coumarate:CoA ligase
comp87029_c0_seq16	0.02	0.66	+	39.10	SapurV1A,1384s0010,1,p	4-coumarate:CoA ligase
comp87029_c0_seq21	0.12	3.64	+	31.44	SapurV1A,1384s0010,1,p	4-coumarate:CoA ligase
comp87029_c0_seq17	0.15	3.20	+	21.42	SapurV1A,1384s0010,1,p	4-coumarate:CoA ligase
*CHS*						
comp92924_c0_seq6	0.02	4.89	+	265.38	SapurV1A,0820s0070,1,p	Chalcone synthase
comp92851_c3_seq6	0.08	13.40	+	176.87	SapurV1A,0056s0660,1,p	Chalcone synthase
comp92851_c3_seq1	0.18	10.77	+	61.19	SapurV1A,0820s0080,1,p	Chalcone synthase
comp92924_c0_seq2	0.06	3.45	+	59.52	SapurV1A,0820s0070,1,p	Chalcone synthase
comp92924_c0_seq1	1.29	65.97	+	51.18	SapurV1A,0056s0640,1,p	Chalcone synthase
comp92851_c3_seq5	4.59	214.20	+	46.67	SapurV1A,0820s0070,1,p	Chalcone synthase
comp92924_c0_seq9	0.17	7.59	+	45.89	SapurV1A,0056s0640,1,p	Chalcone synthase
comp92924_c0_seq3	4.61	207.87	+	45.07	SapurV1A,0820s0070,1,p	Chalcone synthase
comp92851_c3_seq2	4.07	175.83	+	43.18	SapurV1A,0056s0640,1,p	Chalcone synthase
comp92851_c3_seq4	7.84	313.55	+	40.01	SapurV1A,0820s0080,1,p	Chalcone synthase
comp92924_c0_seq8	1.73	66.37	+	38.44	SapurV1A,0056s0640,1,p	Chalcone synthase
comp92924_c0_seq10	0.08	2.72	+	34.45	SapurV1A,0056s0640,1,p	Chalcone synthase
comp92924_c0_seq4	4.96	152.70	+	30.78	SapurV1A,0820s0070,1,p	Chalcone synthase
comp92924_c0_seq11	0.02	0.34	+	20.61	SapurV1A,0056s0640,1,p	Chalcone synthase
comp92924_c0_seq7	0.02	0.38	+	20.14	SapurV1A,0056s0660,1,p	Chalcone synthase
comp75793_c0_seq1	6.38	111.69	+	17.51	SapurV1A,0542s0090,1,p	Chalcone synthase
comp92924_c0_seq5	0.09	1.22	+	12.93	SapurV1A,0056s0640,1,p	Chalcone synthase
*CHI*						
comp95229_c1_seq6	0.22	2.86	+	13.17	SapurV1A,0245s0030,1,p	Chalcone-flavonone isomerase
comp93564_c1_seq7	0.70	0.33	−	2.13	SapurV1A,0130s0520,1,p	Chalcone-flavanone isomerase
*F3H*						
comp93715_c0_seq5	0.93	28.97	+	31.12	SapurV1A,1567s0010,1,p	Flavanone 3-hydroxylase
comp93715_c0_seq2	0.87	26.63	+	30.44	SapurV1A,1567s0010,1,p	Flavanone 3-hydroxylase
comp93715_c0_seq4	1.35	12.11	+	9.00	SapurV1A,1567s0010,1,p	Flavanone 3-hydroxylase
*FLS*						
comp93365_c1_seq19	1.22	0.16	−	7.54	SapurV1A,1087s0040,1,p	Flavonol synthase
comp94406_c0_seq5	18.33	12.03	−	1.52	SapurV1A,1595s0040,1,p	Flavonol synthase
comp94406_c0_seq24	19.86	14.07	−	1.41	SapurV1A,1595s0040,1,p	Flavonol synthase
*F3′5′H*						
comp46256_c0_seq1	0.04	1.03	+	25.07	SapurV1A,1430s0020,1,p	Flavonoid 3',5'-hydroxylase
F3′H						
comp84735_c0_seq3	1.07	11.53	+	10.81	SapurV1A,0426s0030,1,p	Flavonoid 3'-hydroxylase
comp84735_c0_seq1	0.11	1.16	+	10.64	SapurV1A,0426s0030,1,p	Flavonoid 3'-hydroxylase
*DFR*						
comp77300_c0_seq1	0.03	0.86	+	33.95	SapurV1A,0188s0360,1,p	Dihydroflavonol 4-reductase
comp77300_c0_seq2	3.03	90.20	+	29.77	SapurV1A,0006s0390,1,p	Dihydroflavonol 4-reductase
comp81865_c0_seq1	1.55	3.12	+	2.02	SapurV1A,5526s0010,1,p	Dihydroflavonol-4-reductase
comp90439_c0_seq11	1.10	0.43	−	2.57	SapurV1A,1769s0020,1,p	Dihydroflavonal-4-reductase
*ANR*						
comp85880_c0_seq3	0.02	1.15	+	46.15	SapurV1A,0028s0410,1,p	Anthocyanidin reductase ANR1-1
comp85880_c0_seq4	0.72	18.48	+	25.61	SapurV1A,0028s0410,1,p	Anthocyanidin reductase ANR1-1
comp85880_c0_seq11	7.28	135.20	+	18.58	SapurV1A,0028s0410,1,p	Anthocyanidin reductase ANR1-1
comp85880_c0_seq9	0.27	4.19	+	15.80	SapurV1A,0028s0410,1,p	Anthocyanidin reductase ANR1-1
comp85880_c0_seq6	0.10	1.59	+	15.69	SapurV1A,0028s0410,1,p	Anthocyanidin reductase ANR1-1
comp85880_c0_seq8	0.11	1.16	+	10.52	SapurV1A,0028s0410,1,p	Anthocyanidin reductase ANR1-1
*ANS*						
comp89298_c0_seq1	1.88	79.16	+	42.18	SapurV1A,0260s0310,1,p	Anthocyanidin synthase
comp89298_c0_seq2	0.16	3.30	+	20.57	SapurV1A,0260s0310,1,p	Anthocyanidin synthase
*LAR*						
comp86991_c0_seq2	0.21	2.16	+	10.30	SapurV1A,4044s0010,1,p	Leucoanthocyanidin reductase

Twenty-four transcripts encoding ABC transporter proteins were differentially expressed, 20 of which were up-regulated in leaves from treated plants (Table 3). Additionally, three transcripts of the vacuolar cation/proton exchanger 2 (CAX2), encoding the same two proteins differentially expressed in the roots (SapurV1A.0338s0120.x.p and SapurV1A.1071s0020.x.p), were also up-regulated in treated leaves. In contrast to this, two transcripts encoding two vacuolar cation/proton exchanger (CAX) proteins (SapurV1A.0001s0630.x.p and SapurV1A.0077s0070.x.p) were down-regulated in response to treatment. Two transcripts encoding proteins previously associated to cadmium presence were also up-regulated in treated leaves; a cadmium induced protein (SapurV1A.0051s0540.x.p) (1.85 fold higher) and a cadmium resistance protein (SapurV1A.0227s0040.x.p) (2.70 fold higher). Distinctive from root gene expression, two transcripts encoding phytochelatin synthetases proteins (SapurV1A.0323s0100.x.p and SapurV1A.0546s0010.x.p) were down-regulated (2.25 and 8.63 fold lower respectively) in leaves of arsenic treated plants. In terms of plant communication, differentially expressed genes involved in ethylene biosynthesis were more abundant in leaves of arsenic treated plants: four transcripts encoding aminocyclopropane-1-carboxylate oxidase (ACO) (three proteins SapurV1A.1406s0060.x.p, SapurV1A.0666s0130.x.p and SapurV1A.0285s0010.x.p) were up-regulated in treated leaves while one (SapurV1A.0874s0100.x.p) were down-regulated.

**Figure 3:**
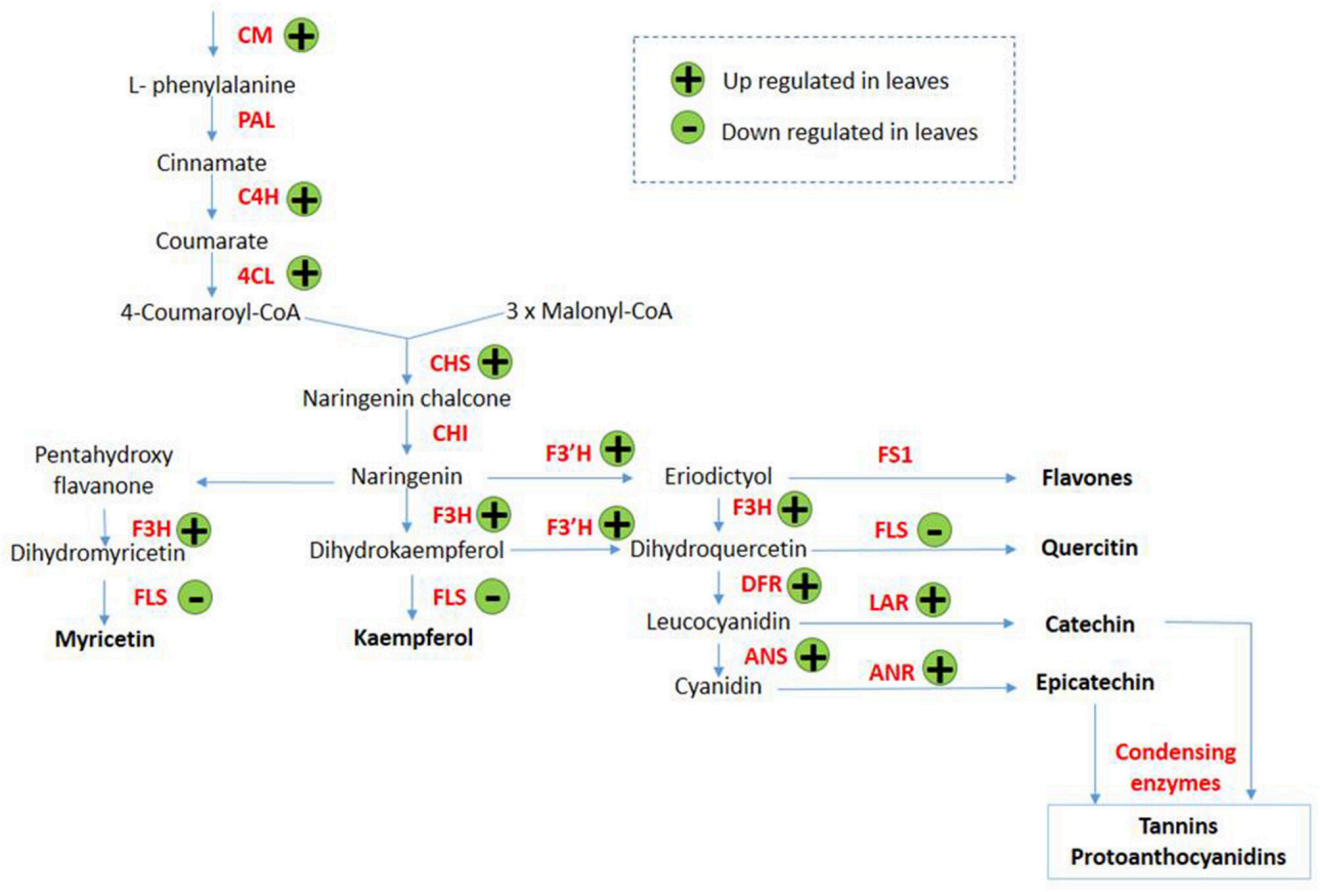
Proposed phenylpropanoid/flavonoid pathway expression alterations in *Salix purpurea* leaves exposed to arsenic. (+) up-regulation and (−) down-regulation represented within differentially expressed genes (having a posterior probability of being differentially expressed >0.95). CM, Chorismate mutase; PAL, phenylalanine ammonialyase; C4H, cinnamate 4-hydroxylase; 4CL, 4-coumarate-CoA ligase; CAD, cinnamyl alcohol dehydrogenase; CCR, cinnamoyl CoA reductase; CHS, chalcone synthase; CHI, chalcone isomerise; F3H, flavanone 3-hydroxylase; F3'H, flavonoid 3′-hydroxylase; FS1, flavone synthase; FLS, flavonol synthase; DFR, dihydro-flavonol 4-reductase; LAR, leucoanthocyanidin; ANS, anthocyanidin synthase; ANR, anthocyanidin reductase. Adapted from Winkel-Shirley (2002) and Anderson and Chapple (2014).

Transcripts encoding enzymes regulating the phenylpropanoid and flavonoid pathways were differentially expressed in leaves in response to arsenic treatment. The first steps of these pathways are shared and involve chorismate mutase (SapurV1A.0372s0140.x.p), whose transcripts were up-regulated in treated leaves (4.56 fold higher) (Table 3 and Figure 3). A subsequent key enzyme in the pathway is phenylalanine ammonialyase (PAL), which was not identified as differentially expressed, but whose action generates the substrate for the next enzyme shared by these pathways; cinnamate-4-hydroxylase (SapurV1A.0215s0280.x.p), encoded by a single differentially expressed transcript here which was up-regulated (10.76 fold) in arsenic treated leaves. Five transcripts encoding coumarate CoA ligase (SapurV1A.1384s0010.x.p) were all up-regulated (between 21 and 91 fold higher) in treated leaves as were all of 15 transcripts encoding chalcone synthase (CHS). Three out of these 15 CHS transcripts were the three most abundant transcripts found in leaves (Table 3, Figure 3 and Supplementary File 1). The subsequent enzyme downstream of CHS, chalcone isomerase (CHI), had one down-regulated transcript and one upregulated transcript (encoding SapurV1A.0130s0520.x.p and SapurV1A.0245s0030.x.p respectively) in response to treatment in leaves. Six transcripts encoding Flavanone-3β-hydroxylase (F3H) production were differentially expressed during arsenic treatment; three (encoding SapurV1A.1567s0010.x.p) were up-regulated in treated leaves and 3 were down-regulated (encoding SapurV1A.1087s0040.x.p and SapurV1A.1595s0040.x.p). Two Flavonoid 3′-hydroxylase (F3′H) transcripts (encoding SapurV1A.0426s0030.x.p) were also up-regulated in treated leaves (10.64 and 10.81 fold higher respectively) as well as transcript encoding Flavonoid 3′–5′ hydroxylase (F3′5′H) (SapurV1A.1430s0020.x.p; 25.07 fold higher). Flavone synthase (FS1) wasn't differentially expressed due to arsenic treatment in leaves but three flavonol synthase (FLS) transcripts (encoding SapurV1A.1595s0040.x.p and SapurV1A.1087s0040.x.p) were all down-regulated in treated leaves. Three dihydroflavonol-4-reductase (DFR) transcripts (encoding SapurV1A.0188s0360.x.p, SapurV1A.0006s0390.x.p and SapurV1A.5526s0010.x.p) were up-regulated in treated leaves (33.95, 29.77, and 2.02 fold higher respectively), including one expressed in extraordinarily high abundance in treated plants (90.20 FPKM), while one was down-regulated (SapurV1A.1769s0020.x.p) (2.57 fold lower).

Transcripts of key enzymes regulating the production of anthocyanins were found to be affected by arsenic stress. Two anthocyanidin synthase (ANS) transcripts (encoding SapurV1A.0260s0310.x.p) were up-regulated in arsenic treated leaves (respectively 42.18 and 27.57 fold higher) as was a transcript encoding leucoanthocyanidin reductase (LAR) (SapurV1A.4044s0010.x.p; 10.30 fold higher). All six transcripts encoding the final enzyme for flavan-3-ol production, anthocyanidin reductase (ANR; SapurV1A.0028s0410.x.p), were all up-regulated in leaves of arsenic treated plants.

#### Non-plant gene expression

A total of 67 transcripts annotated from organisms other than plants were differentially expressed in response to arsenic treatment: three transcripts in leaves, three in stems and 61 in roots (Supplementary File 1). Only six of these transcripts were up-regulated due to arsenic treatment with the vast majority down-regulated. The majority of down-regulated sequences were annotated as originating from Amoebozoa, Metazoa and fungi (including an unknown *Fomitiporia mediterranea* transcript expressed at high abundance in controls, 28.64FPKM). Of the five transcripts up-regulated in response to arsenic treatment, three were annotated from the fresh water rotifer *Philodina roseola*.

## Discussion

### Response to arsenic and arsenic accumulation

Each of the three arsenic concentrations applied hydroponically to juvenile willow trees had significant detrimental effects on plant development, showing the extremity of arsenic toxicity is similar to that seen in other generally high-tolerance species such as, *Pteris vittata* (Chinese brake), *Typha latifolia*, and *Oryza sativa* (rice) (Dushenko et al., 1995; Dai et al., 2013; Sahoo and Kim, 2013). The majority of up-taken arsenic accumulated in the roots. Little or no arsenic was translocated to the aboveground tissues at the lowest concentration of 5 mg/L arsenic but translocation was observed in the most concentrated condition of 100 mg/L. These findings support previous results indicating willows have the capability to translocate arsenic from roots to above ground organs (Sylvain et al., 2016) but could be the cause or result of general plant dysfunction at this high concentration. Although arsenic was not detected at the lowest arsenic treatment, small amounts below the detection limit could potentially have been translocated from the growth solution to the leaves. The impact of the two higher arsenic concentrations, 30 and 100 mg/L, on tree development was so severe that measurements such as chlorophyll content and stomatal conductance could not be taken. In contrast to this, chlorophyll content and stomatal conductance in plants exposed to 5 mg/L arsenic could be measured and were not significantly different to control plants (Figure 1). As there was no variation in chlorophyll content and transpiration rate, it seems likely that the any impact to development between 0 and 5 mg/L are independent of photoassimilation rates. Due to plant tolerance of arsenic at 5 mg/L, this treatment level was selected as suitable for further genetic investigations of successful tolerance mechanisms.

### Differential gene expression in roots

While arsenic uptake was clearly measured in treated willows, the chemical form of intracellular arsenic is hard to qualitatively assess. In the environment, arsenic exists predominantly in two forms; arsenate (As^V^) and arsenite (As^III^) depending on pH, redox conditions and potential ligands (Tripathi et al., 2007; Zhao et al., 2009). At the soil/root interface, arsenate can enter roots using phosphate transporters, while arsenite can be taken up by aquaporins (Bhattacharjee et al., 2008). As arsenate was added to the system it is perhaps unsurprising that the phosphate transporter PHO1 was upregulated. Quaghebeur and Rengel (2004) suggested that uptake inhibition is a key mechanism for successful tolerance. This was demonstrated by Meharg and Macnair (1992) in *Holcus lanatus*, an arsenic tolerant perennial grass, which inhibit phosphate transport by down-regulation of associated genes, simultaneously reducing arsenate uptake and damage to tissues. If this paradigm of arsenic exclusion tolerance is accepted, then *Salix purpurea* would not be considered tolerant as uptake occurs. Thus, *Salix pupurea* not being an exclusion tolerance plant could explain increases in arsenic toxicity endurance in its tissues, through a necessity for a more effective detoxification than exclusion tolerant plants.

Aquaporins, in particular NIP1.1, were also differentially expressed in roots of arsenic treated plants. NIP1.1 has previously been reported as mediating arsenite entry into roots (Kamiya et al., 2009) raising the possibility that arsenate reduction could have occurred prior to its entry into the roots. Another aquaporin TIP2.1 was down-regulated in treated roots, suggesting that NIP1.1 transcriptional response is specific to arsenic treatment as opposed to generalized stress-related aquaporin upregulation. As arsenite is a structural analog of silicic acid, arsenite could also enter root epidermal cells using silicon transporter Lsi1 (low silicon 1), a homolog of the NIP2.1 aquaporin, which has been shown to be a major entry point of arsenite and methylated arsenite in the roots of rice (Li et al., 2009). The silicon transporter was not, however, differentially expressed in roots here, suggesting either a potential point of divergence in tolerance strategies between the two crops or that the transport activity is not related to transcript abundance.

Reduction of arsenate to arsenite shortly following uptake into root cells could occur via either a non-enzymatic reaction with glutathione (GSH) or by an arsenate reductase, such as a tyrosine phosphatase (Dhankher et al., 2002; Bleeker et al., 2006). Interestingly, one tyrosine phosphatase was up-regulated in treated plants (SapurV1A.0142s0310.x.p) while another was down-regulated (SapurV1A.0243s0430.x.p). This observation gives more weight to the hypothesis that SapurV1A.0142s0310.x.p expressed in treated plants (highly divergent from SapurV1A.0243s0430.x.p) could be involved in arsenate reduction to arsenite. Genes involved in GSH production, the non-enzymatic reduction pathway, were up-regulated in response of arsenic treatment, allowing the potential for both mechanisms of arsenate reduction to be considered. Especially given that glutathione synthetase (GS) was one the most abundant of all transcripts in treated plants (Supplementary File 1).

One of the potential fates of arsenite, if uptaken directly, could be extrusion, or efflux back into the soil. The principal mechanism for arsenite efflux is thought, again, to be the bidirectional channel Lsi1 (Zhao et al., 2010). This phenomenon has been reported in several plants species (Xu et al., 2007; Su et al., 2009; Zhang et al., 2009) suggesting that arsenite extrusion could be a general, and understandable, arsenic detoxification process in plants. As *Salix* potentially doesn't use Lsi1 as a means of detoxification, tolerance could rely on other extrusion actors such as the previously described NIP aquaporin (Bienert et al., 2008) or mechanisms relying on vacuolar transporters such as ABC or CAX.

Methylation of arsenic often occurs to facilitate the detoxification process in fungi and bacteria (Clemens and Ma, 2016) and some evidence has been reported in tolerant plants species (Wu et al., 2002; Norton et al., 2008), although rhizospheric interactions have the potential to confound analyses (Lomax et al., 2012). Although the pathway for arsenic methylation in plants is unknown, S-adenosyl methionine (SAM)-dependent methyltransferase could be a potential enzymatic mechanism (Zhao et al., 2009). Although numerous SAM-dependent methyltransferase transcripts were significantly upregulated in treated plant, a roughly equal number were downregulated, demonstrating the complexity of these regulatory processes.

In the cytosol of root cells, arsenite toxicity originates from reacting with sulfhydryl groups to disrupt cell function. CAX2, a transporter involved in cadmium transfer to vacuoles (Koren'kov et al., 2007), has not been directly implicated in arsenite transport. Interestingly, CAX2 expression was highly increased in treated plants, indicating a potential role in direct arsenite vacuolar loading. However, in order to reduce this reactivity, arsenite is thought to predominantly be complexed to glutathione or phytochelatins (PCs) (Pickering et al., 2000). The transient complexes have low stability and need to be transferred to the vacuole where acidic conditions improve stability (Schmöger et al., 2000). Numerous members of the ATP-binding cassette (ABC) transporter family, potentially transporting arsenite-PC complex into vacuoles (Song et al., 2010, 2014) were upregulated due to treatment, however, a greater number were downregulated (Table 1). Future research needs to establish functional differences between these important ABC transporters in non-model tolerant crops before this complex expression pattern can be resolved. Unsurprisingly, phytochelatin production itself is considered a likely critical factor of detoxification and endurance of metal contamination in many plants species (Hartley-Whitaker et al., 2001; Verbruggen et al., 2009). Regulation of PC biosynthesis begins with the production of glutathione (GSH) (Grill et al., 1989). Glutathione synthase (GS) was clearly up-regulated in roots of arsenic treated trees, as was glutathione γ-glutamylcysteinyltransferase (phytochelatine synthetase) itself. Interestingly, previous research suggested PCs may not be involved in heavy metal tolerance in *Salix* species (Landberg and Greger, 2004). Our results contradict this and suggest that PC complexing is a mechanism used in *Salix* for arsenic tolerance.

Although hormone responses to treatment are difficult to observe at a transcript level due to the complexity of communication regulatory networks, ACC synthase was upregulated, presenting the potential for ethylene as a means of communication between the roots and stems in response to arsenic exposure.

### Differential gene expression in stems

Gene ontology enrichment from the stem tissue indicated a global reduction in cellular processes due to arsenic treatment. Terms including nucleic acid binding, RNA binding, translation and ribosome protein panther terms were reduced in stems of arsenic treated plants. Aligned with this, downregulation of ribosome expression was a stark pattern in treated stems: four R40s proteins had strongly reduced abundance encoded by the 10 most abundant transcripts in control stems (Table 2, Supplementary File 1). Byrne (2009) linked lower ribosome production, potentially associated to a lower rate of protein production, to a reduced growth. This could be expected given the observed reduction in biomass yield. Additionally, while the up-regulation of ubiquitin ligase activity Panther term due to treatment could represent post-translational protein modification relating to most cellular processes, there is the potential that, in the context of arsenic treatment, this could represent a toxicity coping/amino acid scavenging mechanisms through general increased activity of the ubiquitin proteasome degradation pathway (Figure 2).

Although no arsenic was observed in stems, detection limits are high (>5 mg/L) in relation to what could be physiologically relevant levels and several genes were differentially expressed in stems which indicate a likelihood that there may be arsenic in stems below detection limit. Silicon transporters, such as Lsi1, have the capability to be key xylem loading transporters of MMA (V) (mono-methyl arsenate) and DMA (V) (dimethyl arsenate) for transport to above-ground tissues of plants (Li et al., 2009). Lsi1 was upregulated in the stems of arsenic treated plants (alongside SAM transcripts in roots) so that xylem loading and transport of methylated arsenic to above-ground tissues would be a candidate endurance mechanism. Although, the observed variation in gene expression could be a response to secondary effects of arsenic treatment, such as desiccation or oxidative stress, transport above-ground would be expected as based upon high arsenic concentration applications (30 and 100 mg/L) which, while killing the plants, did contain transported arsenic in above-ground tissues. This is further supported by the up-regulation of the vacuolar transporter CAX2 in response of arsenic treatment, similar to treated roots. The CAX2 protein encoded by the transcript expressed in stems (SapurV1A.0338s0120.x.p) was the same as one of those upregulated in roots (the roots also upregulated a second putative CAX2 protein; SapurV1A.1071s0020.x.p).

Surprisingly, sucrose phosphate synthase (SPS), important for carbon partitioning and in cellulose biosynthesis (Li et al., 2013), were up-regulated in treated plants. This could represent an increase in mobilization of stored glucose in starch due to increased energy or carbon demand, for example: to supplement a reduced rate of photosynthesis due to leaf damage in treated plants (although chlorophyll content and stomatal conductance were unchanged). Another expression pattern which could be associated with reduced biomass yield could be the lower expression of Fasciclin-like arabinogalactan (FLA) transcripts in treated stems which have been shown to contribute to plant stem development and cellulose deposition (MacMillan et al., 2010). Two enzymes involved in callose synthesis were down-regulated during arsenic exposure. This is surprising if *Salix* is indeed not employing an exclusion tolerance strategy as callose synthesis has been reported as an early defense reaction to metal contamination (Jutsz and Gnida, 2015) with deposition hypothesized as a mechanism to reduce metal ion entry via diffusion into cells.

A reduction of expression in genes relating to ethylene signaling in stems of treated plants was distinct from roots. Ethylene is usually produced in substantial amounts in the stem as woody tissue develops (around 6 weeks post-establishment) in regular growth conditions; therefore a reduction in treated plants could be caused by a delay in development of treated plants (Morgan and Drew, 1997). Transcription of genes from the salicylic acid (SA) biosynthesis pathway, often considered as biotic stress response specific in willow, were substantially up-regulated in response to arsenic treatment. This is in agreement with research conducted in *Thlaspi* sp., a nickel (Ni) hyperaccumulator, that demonstrated a role for SA signaling in Ni tolerance (Freeman et al., 2005).

### Differential gene expression in leaves

Transporter activity was the second most represented category in gene ontology enrichment (Panther terms) found in leaves of arsenic treated plants (Figure 2). Up-regulation of transporters due to arsenic treatment might signify the presence of arsenic in leaves. Contrary to this, following the measured entry of arsenic into root cells from soil, signals could have been sent to aboveground tissue in order to prepare organs for potential arsenic toxicity, provoking gene expression patterns indicative of direct arsenic tolerance mechanisms in leaves. Differentially expressed genes in leaves similar to those of stems and/or roots included the phosphate transporter PHO1. While distinct PHO1 proteins were up and down-regulated due to treatment in leaves, the same protein up-regulated in roots was down-regulated in leaves. Although arsenate could drive expression here, the higher transcription of PHO1 in roots could be the consequence of phosphate depravation due to arsenate competition with this physiologically essential compound. Aquaporins are seemingly involved in the treatment response in leaves with NIP, NIP1.1, TIP1, and SIP1 all up-regulated. Similar expression of NIP1.1 in roots and leaves gives some additional weight to the hypothesis of direct arsenite transport. In contrast to TIP2, down-regulated in treated roots, TIP1 was upregulated. As TIP family proteins are not considered arsenic transporters (Zhao et al., 2009), further functional investigation into their potential roles may be fruitful. An unexpected result in leaves was the up-regulation a boron transporter (SapurV1A.0014s0200.x.p) in in response to arsenic treatment. Boron and arsenic belong to the same chemical family, so this may reflect a lack of the specificity indicated from annotation. As observed in roots and stems, CAX2 vacuolar transporters were also differentially expressed and up-regulated in leaves of arsenic treated plants. In contrast to this, CAX1 expression was down-regulated, potentially highlighting a point of distinction between CAX1 and CAX2 (both of which have are reported as cadmium vacuolar transporters) (Baliardini et al., 2015; Zhang et al., 2016).

Genes within the phenylpropanoid pathway and, markedly, those driving flavonoid biosynthesis, were upregulated in leaves of arsenic treated plants. Chalcone synthase (CHS) transcripts were the most abundant of all the differential expression in leaves; moreover, expression of downstream genes within flavonoid biosynthesis which lead specifically to proanthocyanidin (tannins) production were up-regulated due to arsenic treatment. This suggests a potential role for tannins in arsenic metabolism, or as a secondary consequence of arsenic treatment, such as oxidative stress mitigation, in *Salix purpurea*. It has been hypothesized that flavonoids could have a role in metal chelation (Winkel-Shirley, 2002) and previous research has described chelating power of tannins with copper (Cu), zinc (Zn), cobalt (Co), and aluminum (Al) (McDonald et al., 1996; Kainja et al., 1998). Davis et al. (2001) also proposed that in hyperaccumulators, tannins may function as metal-binding compounds, allowing for some potential overlap of metal stress tolerance mechanisms between hyperaccumulators and *Salix purpurea*. This high level of upregulation of genes toward tannin biosynthesis is interesting in the context of recent research published by Gonzalez et al. (2015) that suggested cross-tolerance of contamination treated *Salix* against biotic stress in the form of a herbivorous arthropod. The agency of this cross-tolerance was hypothesized as tannin upregulation due to organic hydrocarbon contamination in soil, as tannins are recognized as highly unpalatable to many herbivorous arthropods. As the similar response of tannin upregulation in leaves here was induced by arsenic contamination, it seems likely to be in response to secondary toxicity conditions in the plant, such as oxidative stress which would be commonly induced by both contaminants, as opposed to a direct response to the presence of arsenic in the leaves. Although treated plants had reduced biomass yields within this hydroponic system, willows cultivated in field at low concentrations of trace elements can often maintain relatively high biomass yields. One mechanism potentially explaining this could be such cross-tolerance mechanisms whereby increased leaf tannin concentrations confer an advantage by reducing arthropod predation in field conditions (which would not be observed within a hydroponic cultivation system).

### Differential gene expression of foreign organisms associated with *Salix* tissue

Differentially expressed transcripts from putative non-plant organisms were observed in the assembled *de novo* transcriptome, which was possible due to the unconstrained annotation procedure and important as to assess as such gene expression can both technically (Brereton et al., 2016) and biologically (Gonzalez et al., 2015) confound transcriptome data analysis. Although “foreign” differential gene expression was observed, the numbers of identified contigs (67) were minor and we, therefore, do not consider the results of the plant gene expression to likely be confounded through metatranscriptomic or microbiome interactions. Moreover, although the presence of the species (or close relatives) that these transcripts were best annotated from was not directly confirmed, the overall expression pattern observed might be insightful regarding the molecular processes in the system. Within these sequences, amoeba, fungi, metazoan, and proteobacteria expression was identified (most represented within roots, 91%) and was almost comprehensively downregulated in treated plants. Two of the most represented genera in these transcripts from roots were *Dictyostelium* (a slime mold) and *Acanthamoeba*, both bacterivores common to fresh water and soil, and perhaps unsurprising inhabitants of a hydroponic system. Whilst these organisms were presumably not arsenic tolerant, resulting in the down-regulation of their differentially expressed genes, three differentially expressed genes annotated as coming from *Philodina roseola* (isolated from roots and stems) were all upregulated in arsenic treated plants. Intriguingly *P. roseola* has been shown to decrease cadmium metal presence by 76% at similar concentrations (slightly higher 10 mg/L) within investigations of the species as an urban wastewater bioremediator (Rehman et al., 2008). Another one of very few upregulated transcripts, from the *Bacteroides* sp. 2_1_33B, was a putative phosphatase family protein. Many microbial communities have abilities for arsenic tolerance and Tiwari et al. (2016) recently discovered an arsenic resistant endophytic bacteria from *Pteris vittata* roots capable of arsenate reduction. Bacterial arsenate reduction using phosphatase is well characterized (Zegers et al., 2001) and the up-regulated phosphatase in arsenic treated *Salix* roots may indeed represent a similar arsenic tolerant endophyte, although bacterial isolation and assessment of arsenate reduction activity is needed to confirm this hypothesis.

## Conclusion

While not considered as a metal hyperaccumulating species, *Salix purpurea* appears to be able to react to arsenic using molecular mechanisms usually observed in tolerant hyperaccumulator species as opposed to utilizing contaminant exclusion tolerance strategies. Based on the results reported here, we also suggest that willows respond to arsenic contamination by inducing the biosynthesis of phenylpropanoids that may culminate with the increased production of tannins. This non-exclusion physiological response to metal contamination, coupled with high biomass yields, makes willow sp. an attractive option for contaminated site phytoremediation and, importantly, selection toward improved arsenic accumulation capabilities.

## Author contributions

FP, SJ, and ML designed the study. AY performed the plant growth trials and sample preparation. AY, EG, and NB analyzed the data and drafted the manuscript. All authors edited the manuscript and approved the final manuscript.

### Conflict of interest statement

The authors declare that the research was conducted in the absence of any commercial or financial relationships that could be construed as a potential conflict of interest.
